# Interactions between 2′-fluoro-(carbamoyl­pyridinyl)deschloroepibatidine analogues and acetylcholine-binding protein inform on potent antagonist activity against nicotinic receptors

**DOI:** 10.1107/S2059798322000754

**Published:** 2022-02-21

**Authors:** Renata V. Bueno, Samuel Davis, Alice Dawson, Pauline W. Ondachi, F. Ivy Carroll, William N. Hunter

**Affiliations:** a Division of Biological Chemistry and Drug Discovery, School of Life Sciences, University of Dundee, Dundee DD1 5EH, United Kingdom; b Research Triangle Institute, PO Box 12194, Research Triangle Park, Durham, NC 27709, USA

**Keywords:** acetylcholine-binding protein, biolayer interferometry, crystal structure, epibatidine derivatives, ligand-gated ion channels, nicotinic acetylcholine receptors

## Abstract

Low-nanomolar binding constants were recorded for a series of six 2′-fluoro-(carbamoylpyridinyl)deschloroepibatidine analogues with acetylcholine-binding protein (AChBP). The crystal structures of three complexes with AChBP reveal details of molecular recognition in the orthosteric binding site and imply how the other three ligands bind. Comparisons exploiting AChBP as a surrogate for α4β2 and α7 nicotinic acetylcholine receptors (nAChRs) suggest that the key interactions are conserved. The ligands interact with the same residues as the archetypal nAChR agonist nicotine yet display greater affinity, thereby rationalizing their *in vivo* activity as potent antagonists of nicotine-induced antinociception. An oxyanion-binding site is formed on the periphery of the AChBP orthosteric site by Lys42, Asp94, Glu170 and Glu210. These residues are highly conserved in the human α4, β2 and α7 nAChR sequences. However, specific sequence differences are discussed that could contribute to nAChR subtype selectivity and in addition may represent a point of allosteric modulation. The ability to engage with this peripheral site may explain, in part, the function of a subset of ligands to act as agonists of α7 nAChR.

## Abstract 2

The binding of a series of epibatidine derivatives to acetylcholine-binding protein was investigated using biolayer interferometry. The structures of three complexes inform discussion on the biological implications for interactions with nicotinic acetylcholine receptor subtypes, which are important targets for control of pain.

## 1. Introduction

**Figure 1:**
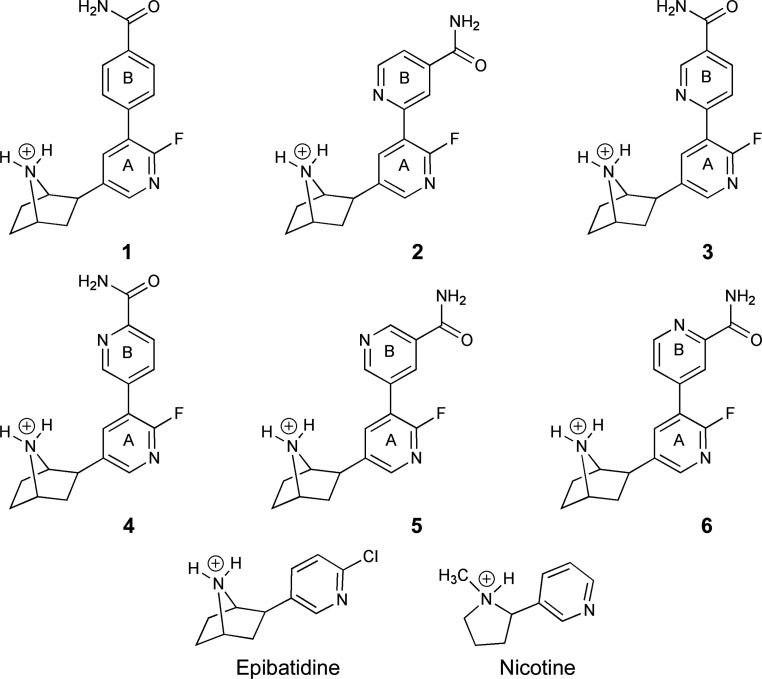
The structures of epibatidine, nicotine and analogues **1**–**6** used in this work. In all cases the protonated form, which is most likely under physiological conditions, is shown. The first aromatic ring after the azabicycloheptane (a fluoropyridine) is labelled A and the second ring labelled B is a benzylcarboxamide in compound **1** and a pyridinecarboxamide in compounds **2**–**6**.

Nicotinic acetylcholine receptors (nAChRs) are cation-selective pentameric ligand-gated ion channels (pLGICs) gated by the neurotransmitters acetylcholine and choline. They are also the targets of non-endogenous molecules, with the best known being the archetypal agonist nicotine. The availability of a variety of subunits enables neuronal nAChRs to assemble in different homopentameric or heteropentameric combinations (Gotti *et al.*, 2009 ▸; Taly *et al.*, 2009 ▸; Bertrand & Terry, 2018 ▸). The most common types in the mammalian central nervous system are an α4β2 combination, which has a high affinity for nicotine, and the α7 homomer (Taly *et al.*, 2009 ▸). The pLGIC family presents a number of therapeutic targets for neurological conditions; specifically, nAChR subtypes are key targets for the development of compounds with use in the treatment of nicotine addiction and also of pain (Bertrand *et al.*, 2015 ▸; Dineley *et al.*, 2015 ▸; Bagdas *et al.*, 2018 ▸) The discovery of epibatidine [(1*R*,2*R*,4*S*)-2-(6-chloro-3-pyridinyl)-7-azabicyclo[2.2.1]heptane; Fig. 1 ▸], a highly potent but relatively nonselective agonist of nAChR that displays powerful non-opiate-mediated antinociceptive effects, elicited great excitement (Spande *et al.*, 1992 ▸; Traynor, 1998 ▸). A serious liability due to toxicity rules out therapeutic use; nevertheless, with high ligand efficiency and potency the compound has provided a basis for the development of analogues that target nAChR (Spang *et al.*, 2000 ▸; Carroll, 2004 ▸; Mu *et al.*, 2006 ▸; Ondachi *et al.*, 2016 ▸). One study focused on the position of the chloropyridine ring N atom and resulted in conversion from an agonist to an antagonist profile (Spang *et al.*, 2000 ▸).

Our interest centres on a series of 2′-fluoro-(carbamoyl­pyridinyl)deschloroepibatidine analogues (compounds **1**–**6**; Fig. 1 ▸), characterized as having high affinity (*K*_i_ < 1 n*M*) for α4β2 nAChR, that display differing degrees of subtype selectivity and novel pharmacological effects compared with epibatidine (Ondachi *et al.*, 2016 ▸). Electrophysiological measurements of ion-channel activity indicated that compounds **1**–**6** have little or no agonist activity on recombinant α4β2 or α3β4 nAChRs. In contrast, the parent compound epibatidine is a full agonist. The compound set demonstrated antagonist activity on α4β2 and α3β4 nAChRs. However, puzzling effects were observed on the homomeric α7 nAChR. Compounds **1** and **3** act as mixed partial agonists of α4β2, whilst compound **2** is only an antagonist of α7 nAChR, with no agonist properties detected. Furthermore, compounds **4** and **5** were selective for α4β2 over α7, and *in vivo* studies indicated that these analogues all antagonize the antinociceptive action of nicotine with, in the case of compound **2**, a potency approaching that of varenicline, a well studied partial agonist of α4β2 receptors and a full agonist of α7 nAChR (Ondachi *et al.*, 2016 ▸). The differing actions at nAChR subtypes are perplexing and we sought to investigate further.

The biological target nAChRs follow the standard structural arrangement of pLGICs, with five subunits creating a central ion pore. Each subunit possesses an extracellular domain (ECD) followed by four transmembrane α-helices and intracellular contributions from the inter-helical segments (Taly *et al.*, 2009 ▸; Bertrand & Terry, 2018 ▸). The orthosteric binding site, where agonists and competitive antagonists bind, is created by contributions from the ECD at the interface between two subunits. One subunit contributes the principal (+) face, which is formed of three loops known as A, B and C. On an adjacent subunit, loops D, E, F and G comprise the complementary (−) side of the binding site (Sixma & Smit, 2003 ▸; Corringer *et al.*, 2012 ▸; daCosta & Baenziger, 2013 ▸; Nys *et al.*, 2013 ▸; Sauguet *et al.*, 2015 ▸). Acetylcholine-binding protein, which is found in the cholinergic synapse of gastropods including *Aplysia californica* (*Ac*AChBP), shares 20–25% sequence identity with the ECD of nAChR sequences. In *Ac*AChBP, 44 residues are involved in the orthosteric binding site and the identities of these residues in human nAChRs range from 32% (β2) to 45% (α4 and α7). In addition, AChBP and nAChR display closely related structures and similar ligand-binding properties (Celie *et al.*, 2004 ▸; Hansen *et al.*, 2005 ▸; Lemoine *et al.*, 2012 ▸; Rucktooa *et al.*, 2012 ▸; Shahsavar *et al.*, 2016 ▸). Despite the hugely impressive developments in studies of membrane-bound pLGIC forms, for example the cryo-EM structure of the heteromeric human α4β2 nAChR (Walsh *et al.*, 2018 ▸), *Ac*AChBP remains a valued surrogate for the study of ligand–receptor interactions. This is due to the convenience of working with a stable, soluble protein for which efficient purification protocols exist and where ordered single crystals can be obtained.

We sought to exploit *Ac*AChBP in this manner to investigate the interactions and affinities of epibatidine analogues **1**–**6** (Fig. 1 ▸). Biolayer interferometry (BLI) provided *K*_d_ values, and the crystal structures of three complexes revealed the mode of binding and interactions of compounds **1**–**3**, providing models for complexes with compounds **4**–**6**. Comparisons and modelling using nAChR sequences and structures allowed us to consider the bioactivity of the compound series and to comment on structure–function relationships that may be exploited in the design of new nAChR ligands as chemical tools and/or with therapeutic potential.

## 2. Materials and methods

### 2.1. Recombinant protein production

A gene encoding *Ac*AChBP with a C-terminal His_6_ tag was cloned into the pFastBac1 vector (Thermo Fisher). The amino-acid sequence was derived from the *A. californica* genome (https://www.broadinstitute.org/aplysia/aplysia-genome-project) and is similar to UniProt entry Q8WSF8 except that two alanine residues are replaced by Val60 and Val155. This construct was expressed using the Bac-to-Bac system (Thermo Fisher) in *Spodoptera frugiperda* (*Sf*9) cells maintained in shaker flasks at 27°C using Insect-XPRESS medium (Lonza) supplemented with 2 m*M* l-glutamine and 100 U ml^−1^ penicillin/streptomycin (Thermo Fisher). The *Ac*AChBP baculovirus was generated through transfection of 500 ng bacmid DNA into adherent *Sf*9 cells at a density of 8 × 10^5^ cells per millilitre using Insect Genejuice reagent (Novagen). Following seven days of incubation at 27°C, the virus was amplified twice in *Sf*9 suspension cultures before being harvested from the medium via centrifugation (1000*g*, 10 min, 4°C).

For protein production, *Sf*9 cells (15 × 10^5^ cells per millilitre) were infected with virus and incubated for approximately 48 h before being separated from the medium by centrifugation (1000*g*, 10 min, 12°C). The recombinant *Ac*AChBP is secreted, so the supernatant was clarified by further centrifugation (4000*g*, 10 min, 12°C), concentrated and exchanged into buffer *A* (50 m*M* Tris–HCl, 250 m*M* NaCl pH 7.5) using a Sartojet system (Satorius) with a Sartocon Slice 10 kDa cutoff microfiltration cassette. *Ac*AChBP was purified by immobilized metal-ion chromatography with Ni^2+^ HisTrap columns (GE Healthcare) and eluted using a linear gradient of buffer *B* (50 m*M* Tris–HCl, 250 m*M* NaCl, 800 m*M* imidazole pH 7.5). Sample purity was assessed using stain-free SDS–PAGE gels (Bio-Rad). The fractions containing *Ac*AChBP were pooled, buffer *B* was exchanged for buffer *A* using 10 kDa centrifugal concentrators and the protein was concentrated to 6 mg ml^−1^.

### 2.2. 2′-Fluoro-(carbamoylpyridinyl)deschloroepibatidine analogues

Compounds **1**–**6** were synthesized as racemic mixtures as described previously (Ondachi *et al.*, 2016 ▸) and then dissolved in DMSO as 100 m*M* stock solutions.

### 2.3. *K*_d_ determination by BLI

The *K*_d_ values of compounds **1**–**6** were determined using an Octet RED system (Forté-Bio). *Ac*AChBP (0.5 mg ml^−1^ in buffer *A*) was immobilized on Ni^2+^–NTA sensors for 600 s, followed by the removal of non-immobilized protein (600 s) and baseline stabilization (120 s). Association and dissociation measurements were obtained at 25°C for six concentrations of each compound (1.7–410 n*M*) diluted in buffer *A* plus 1% DMSO. BLI assays were conducted with a baseline measurement in buffer (60 s), an association measurement in the well containing the compound (120 s) and a dissociation measurement in buffer (120 s). Data processing and analysis were performed with *Octet RED Data Analysis* version 7.1 (Forté-Bio), where the background response of immobilized *Ac*AChBP in buffer was subtracted.

### 2.4. Co-crystallization of *Ac*AChBP with compounds

*Ac*AChBP (4 mg ml^−1^ in buffer *A*) was incubated with 2 m*M* of compounds **1**–**6** for 1 h at room temperature and this mixture was then used in crystallization trials. Compounds **4**–**6** did not yield suitable crystals. Small well formed crystals of *Ac*AChBP with compounds **1**–**3** were obtained using hanging drops consisting of 2 µl complex solution plus 1 µl reservoir solution equilibrated against 800 µl reservoir solution for 24 h at 18°C. The reservoirs consisted of 0.2 *M* NaCl, 0.1 *M* phosphate–citrate pH 4.2 and PEG 8000 at 8% (compound **2**), 10% (compound **3**) or 12% (compound **1**). These conditions allowed us to prepare microseed stocks by transferring crystals to microcentrifuge tubes containing the appropriate reservoir solution, 40% glycerol and a Seed Bead (Hampton Research), and vortexing the sample for 1 min. A cleaned human eyelash was dipped into the seed stock and then passed through freshly assembled crystallization drops. Well ordered multi-faced prisms, with maximum dimensions of about 20 µm, appeared after four days.

### 2.5. Crystallographic analyses

Crystals were harvested using a nylon loop, cryoprotected with reservoir solution adjusted to contain 20% ethane-1,2-diol and then flash-cooled in liquid nitrogen. Diffraction was recorded on Diamond Light Source (DLS) microfocus beamline I24 using a PILATUS 6M-F detector (Dectris) and the images were indexed and integrated using *XDS* (Kabsch, 2010 ▸). The data were scaled using *AIMLESS* (Evans & Murshudov, 2013 ▸) from the *CCP*4 suite (Winn *et al.*, 2011 ▸) and the structures were solved by molecular replacement with *Phaser* (McCoy *et al.*, 2007 ▸), utilizing wild-type protein in complex with strychnine at 1.91 Å resolution (PDB entry 2xys; Brams *et al.*, 2011 ▸) as the model for *Ac*AChBP–**1** and the complex with nicotine at 2.20 Å resolution (PDB entry 5o87; Dawson *et al.*, 2019 ▸) as that for *Ac*AChBP–**2** and *Ac*AChBP–**3**. Ligand models and restraints were generated with the *grade* server (Global Phasing; http://grade.globalphasing.org/cgi-bin/grade/server.cgi). Multiple rounds of automated restrained refinement were completed using *REFMAC*5 (Murshudov *et al.*, 2011 ▸), with manual refinement and model building in *Coot* (Emsley *et al.*, 2010 ▸). The epibatidine analogues were well defined in electron and difference density maps (Supplementary Fig. S1). Asn91 is glycosylated and *N*-acetyl-d-glucosamine (NAG) was modelled onto several subunits. It became clear that additional ligands were present and these were assigned and refined satisfactorily as phosphate, ethane-1,2-diol and oxalate. The latter is likely to be present as a contaminant in PEG 8000 (Fyfe *et al.*, 2010 ▸). Strict noncrystallographic symmetry restraints were applied throughout to *Ac*AChBP–**2** and *Ac*AChBP–**3** but were relaxed towards the end of the refinement for *Ac*AChBP–**1**. Ligand 3D solvent-accessible surface areas were calculated using *AREAIMOL*, with a probe solvent radius of 1.4 Å and 100 surface points per Å^2^. Omit maps were generated by removal of the ligands and bulk-solvent corrections before recalculating the *F*_o_ − *F*_c_ maps presented in Supplementary Fig. S1. Graphics were rendered using the *PyMOL* molecular-graphics system (Schrödinger). Crystallographic statistics are presented in Table 1 ▸. The amino-acid sequences used include human α4 (UniProt code P43681), α7 (P36544) and α2 (P17787) nAChR subtypes.

## 3. Results and discussion

### 3.1. Binding properties

The affinity of *Ac*AChBP for compounds **1**–**6** was assessed by BLI (Table 2 ▸; representative sensorgrams are shown in Supplementary Fig. S2). All compounds displayed *K*_d_ values in the low-nanomolar range, comparable to that of epibatidine (Ondachi *et al.*, 2016 ▸), but show a higher affinity than that seen for nicotine. The ligands can be placed into three groups based on affinity for *Ac*AChBP: compounds **1** and **6** display *K*_d_ values close to 10 n*M*, while the values for compounds **3**, **4**, **5** are around 30 n*M* and that for compound **2** is 60 n*M*. For comparison, the *K*_i_ values determined with a [^3^H]-epibatidine displacement assay against α4β2 nAChR are presented in Table 2 ▸. Consistent with previous *Ac*AChBP ligand-binding studies (Hansen *et al.*, 2005 ▸), the affinities are reduced significantly compared with the subnanomolar levels observed with actual α4β2 nAChR (Ondachi *et al.*, 2016 ▸).

### 3.2. Crystallographic analyses

We attempted to co-crystallize all six compounds with *Ac*AChBP and success with three led to structures of the *Ac*AChBP–**1**, *Ac*AChBP–**2** and *Ac*AChBP–**3** complexes at resolutions of 2.2, 2.4 and 2.5 Å, respectively. The monoclinic crystals obtained in each case are isomorphous, with two pentameric assemblies comprising the asymmetric unit. A high degree of noncrystallographic symmetry was evident and was thus maintained in the refinement calculations, although for *Ac*AChBP-**1**, the highest resolution structure, these restraints were released in the final calculations. The ligands of interest are well defined in the electron density observed in each of the ten binding sites of the structures (Supplementary Fig. S1) and were refined with average *B* factors that were lower than or close to the values noted for their associated subunits (Table 1 ▸). Within each complex, the orientation of the ligand and the pattern of interactions within each binding site are essentially identical and it is only necessary to describe one. Enantiomeric mixtures of compounds **1**–**6** were used, and in the structures with compounds **1**–**3** we tested both forms in modelling to the electron density. The resolution of the crystal structures was insufficient to distinguish (+) and (−) enantiomers or whether a mixture was present, so we used the former to match that of the naturally occurring form of epibatidine, the parent compound. Note that the (+) and (−) forms of epibatidine have very similar binding and biological properties (Mu *et al.*, 2006 ▸; Dallanoce *et al.*, 2012 ▸) and we judge it likely that this also applies to compounds **1**–**6**.

**Figure 2:**
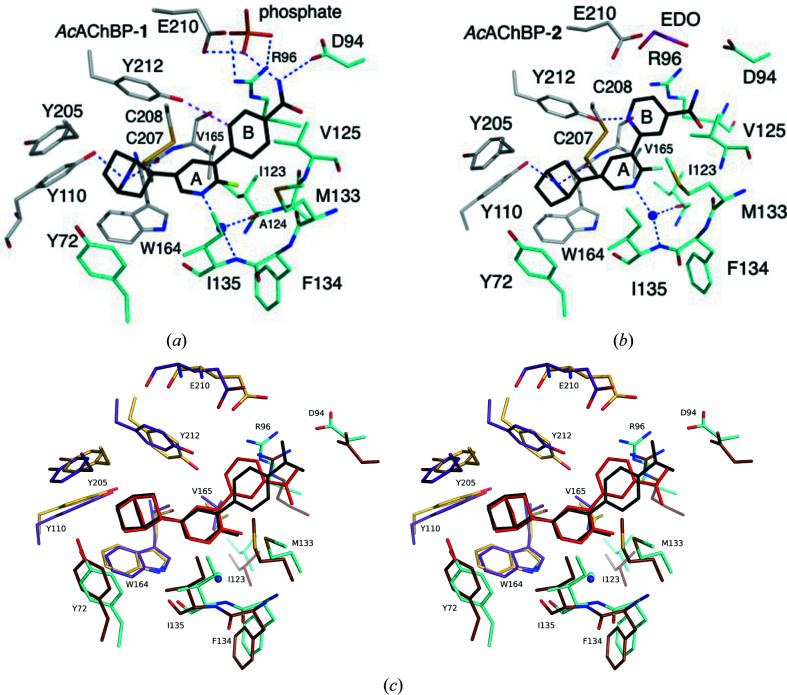
Selected residues involved in interactions with ligands. (*a*) *Ac*AChBP–**1**. (*b*) *Ac*AChBP–**2**. Atoms are coloured using the following scheme: C atoms of the ligands are black, those in the principal subunit are grey and those on the complementary side are cyan, O atoms are red, N atoms are blue, S atoms are yellow and F atoms are green. A marine sphere indicates the position of a conserved water molecule. Blue dashed lines represent potential hydrogen bonds. Rings A and B are labelled. In (*a*) a possible C—H⋯O hydrogen bond is shown as a red dashed line and the P atom is orange, and in (*b*) the C atoms of EDO (ethane-1,2-diol) are pink. Water molecules, main-chain atoms of some residues, for example Cys207-Cys208, and Ala124 in (*b*) are omitted for clarity. (*c*) Stereoview overlay of the two structures. Principal side residues for the complex with compound **1** are shown in violet/purple and those for the complex with compound **2** are shown in yellow/orange. Complementary side residues for the complex with compound **1** are shown in cyan and those for the complex with compound **2** are in brown. The conserved water is shown as a sphere in aquamarine and in dark blue for the complexes with compounds **1** and **2**, respectively. Ligand **1** is coloured black and ligand **2** is coloured red. The coordinates were overlaid using the superpose ligands option in *Coot*.

There are three crystal structures containing epibatidine in the Protein Data Bank (PDB) that are relevant to our study. These are a low-resolution (3.4 Å) complex with *Ac*AChBP (PDB entry 2byq; Hansen *et al.*, 2005 ▸), a 3.2 Å resolution structure with α2 (PDB entry 5fjv; Li *et al.*, 2011 ▸) and a 2.8 Å resolution complex with an α7 chimera (PDB entry 3sq6; Kouvatsos *et al.*, 2016 ▸). Compounds **1**–**3** and epibatidine share the azabicyclo[2.2.1]heptane moiety and this part of the ligand binds in a similar way in all structures, deep in a hydrophobic part of the binding site formed primarily on the principal side. Here, the orthosteric site is dominated by the presence of aromatic residues (Fig. 2 ▸). The protonated amine donates hydrogen bonds to Tyr110 OH and the Trp164 carbonyl group. The position of Tyr110 OH is fixed by hydrogen-bonding interactions with the Ser163 carbonyl and a network of ordered water molecules that form bridges through to Tyr205, and Asp214 (not shown). There are van der Waals interactions with Tyr72, Tyr205, Tyr212, Trp164 and Cys207. The amine is around 4 Å distant from the face of the Trp164 side chain and is positioned to suggest the presence of a cation–π interaction. This interaction is a common and important feature of pLGIC ligand complexes (Taly *et al.*, 2009 ▸; Nys *et al.*, 2013 ▸) and specifically nAChRs (Zhong *et al.*, 1998 ▸). The halogen-substituted pyrimidine ring A, which is common to the series of compounds and epibatidine, is directed towards the complementary side of the orthosteric site, with one face of the aromatic system forming van der Waals interactions with Val165 from the principal side and the other face interacting with Ile135 on the complementary side. A side-on interaction with Cys208 is evident. In the complexes of *Ac*AChBP with compounds **1**, **2** and **3**, the fluorine is directed into a shallow pocket and forms van der Waals interactions with the main chain of Ala124 and Phe134 and the side chains of Val125 and Ile135, all from the complementary side. The pyridine N accepts a hydrogen bond from a water molecule that is at one end of an ordered solvent chain extending to the surface of the protein, forming hydrogen bonds to residues in the orthosteric site, for example to Ile123 O and Trp164 NE1. These parts of the ligands replicate key features found in AChBP–nicotine complex structures; in particular, the presence of an ordered water molecule linking the pyridine N atom to the protein is noted repeatedly (Hansen *et al.*, 2005 ▸; Nys *et al.*, 2013 ▸; Dawson *et al.*, 2019 ▸). The available AChBP–epibatidine complex structures are at low resolution and lack solvent molecules in the binding sites. However, this hydration pattern is strictly conserved across the three structures reported here and is observed in other structures of AChBP complexes (Hansen *et al.*, 2005 ▸; Nys *et al.*, 2013 ▸; Dawson *et al.*, 2019 ▸). It was previously thought that the presence of such an ordered water molecule correlates with agonist activity of the ligand (Nys *et al.*, 2013 ▸), but our structures suggest that such a conclusion does not apply in all cases, a point that we will revisit below.

The structures in the series **1**–**6** have a similar substituent: a phenyl or pyridine ring (termed ring B), with a carboxamide substituent, at the 4′ position of ring A (Fig. 1 ▸). These represent extensions of the epibatidine scaffold. Compound **1** has a phenyl group, and compounds **2**–**6** have pyridines with the N atom at position 2, 3 or 4. A further variation is a carboxamide substituent attached to the pyridine N atom at the *meta* or *para* position. The structures of the complexes of *Ac*AChBP with compounds **1**–**3** all suggest that ring B participates in van der Waals interactions with Arg96, Val125 and Met133 from the complementary side and Cys208 on the principal side (Fig. 2 ▸). In the structure of *Ac*AChBP–**1** the alignment and distances (around 3.3 Å) of the Tyr212 OH group and a carbon on ring B suggest the possibility of a C—H⋯O hydrogen bond (Fig. 2 ▸ *a*). In the complexes with compounds **2** and **3** (Fig. 2 ▸ *b*) the pyridine N atom participates in a hydrogen bond to Tyr212 with distances of around 2.6–3.0 Å.

In the *Ac*AChBP–**1** and *Ac*AChBP–**3** complexes the carboxamide is directed outwards towards bulk solvent. The carbonyl groups participate in water-mediated links to Val125, Thr127 and Ser131 (not shown). The amide groups contribute to a phosphate-binding site, with the oxyanion also coordinated by the side chains of Asp94 and Arg96 on the complementary side and then by Glu170, Glu210, Lys42 and Ser167 from the principal side. This is further detailed below. Phosphate was a component of the crystallization mixture, and the acidic pH used explains why aspartate and glutamate side chains coordinate the oxyanion. In eight binding sites in *Ac*AChBP–**1** and seven in *Ac*AChBP–**3** the amide forms direct hydrogen bonds to Asp94 on the (−) side, perhaps helped by the ordering influence of the oxyanion. Due to the difference in the ring position of the carboxamide substituent in compound **2**, in *Ac*AChBP–**2** the carbonyl group of the ligand is tilted towards loop E on the complementary side, in particular Met133, but ring B is positioned further away from the methionine side chain. An adjustment of the Tyr212 side chain is observed, serving to maintain the hydrogen bond to the ring B N atom. The result is to position the carboxamide in roughly the same position in all three complexes. The pyridine ring B of compound **2** in comparison is posed closer to Tyr212, the position of the amide group is adjusted and our analysis indicated that ethane-1,2-diol, the cryoprotectant, and not phosphate interacts with the amide and engages with the same residues that form the phosphate-binding pocket. A link from the amide of compound **2** to Asp94 is retained but *via* bridging water molecules (not shown). The position of ring B may provide a disruptive block to phosphate binding, and the reduction in van der Waals interactions with Met133 may contribute to compound **2** displaying the lowest affinity for *Ac*AChBP.

**Figure 3:**
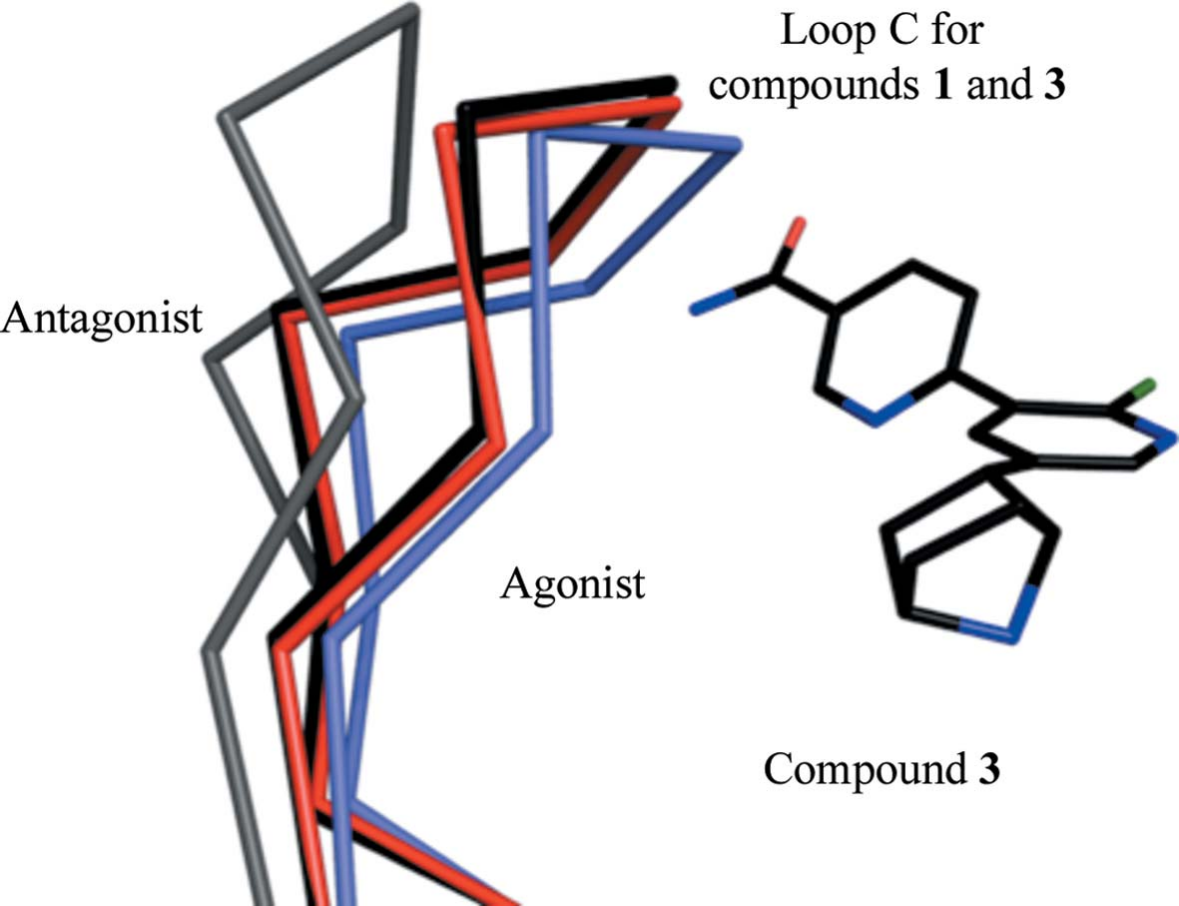
Loop C of *Ac*AChBP adopts different conformations. Ribbon representation of loop C segments with the bound agonist epibatidine (cyan, PDB entry 2byq; Hansen *et al.*, 2005 ▸), antagonist strychnine (grey, PDB entry 5o8t; Dawson *et al.*, 2019 ▸) and the *Ac*AChBP–**1** and *Ac*AChBP–**3** complexes (red and black, respectively). Compound **3** is shown and coloured with the following scheme: C, black; O, red; N, blue; F, dark green. The overlay was calculated using all C^α^ atoms of a single subunit.

Loop C, on the (+) side, contributes significantly to the creation of the orthosteric site and interacts directly with residues on the (−) side. This loop has to present an open conformation to allow ligands to enter the site and then close as the complex forms (Jadey & Auerbach, 2012 ▸). When ligands **1**–**3** occupy the site, loop C closes over them (Fig. 3 ▸). The solvent-accessible surface areas of unbound compounds **1**–**3** are approximately 500 Å^2^, and between 91% and 94% of this surface is buried in the complexes with *Ac*AChBP, with primarily the carboxamide moiety directed towards solvent. Epibatidine is smaller (Fig. 1 ▸), with a solvent-accessible area of 360 Å^2^, and is almost entirely buried (>99%) deep in the binding site, with loop C in the agonist-bound state (Fig. 3 ▸; Spang *et al.*, 2000 ▸).

Compounds **2**, **5** and **6** differ only in the position of the N atom on pyridine ring B. The *Ac*AChBP–**2** complex indicates a displacement of ring B compared with the other structures and we speculate that this might influence the interactions in and around the observed oxyanion-binding site. The placement of the ring B N atom in compound **6** would position a hydrogen-bond acceptor in an ideal position to participate in formation or organization of the anion-binding site. Using an overlay of complexes with compounds **1** and **2** as a template, the N atom in ring B would be about 2.4 Å from a phosphate in compound **6** and around 2.9 Å in compound **5**.

**Figure 4:**
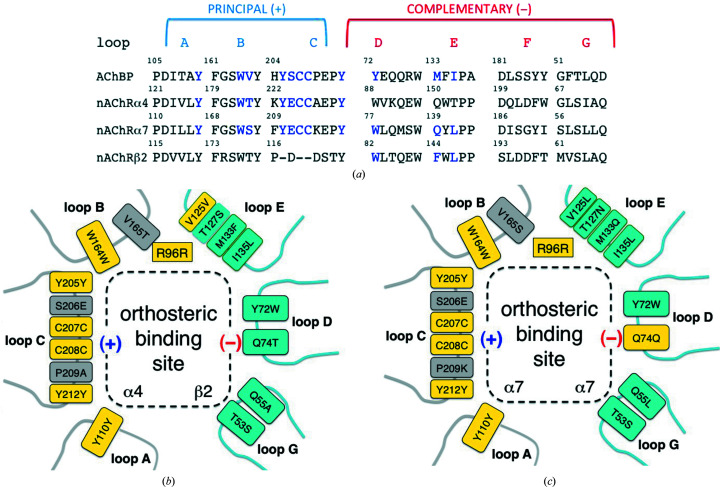
(*a*) Alignment of selected sequence segments that form the orthosteric binding sites in *Ac*AChBP and α4, α7 and β2 nAChRs. Loops are labelled and are split into principal (+) and complementary (−) sides. Residues coloured blue are key and are discussed in the text. Val125 and Thr127 of *Ac*AChBP are in the N-terminal section of loop E but are left out for clarity. (*b*) A schematic of the orthosteric binding site listing the key residues of *Ac*AChBP and the corresponding residues in the α4(+)β2(−) nAChR heteromeric site. Loop F is out of range of the ligands discussed in this work and has been omitted. Arg96 is included given its role in binding the anion (see text). Boxes are coloured yellow to highlight strict conservation, grey for the (+) side and cyan for the (−) side. (*c*) Schematic with the same format for the α7 nAChR homomeric site.

Comparisons of the *Ac*AChBP complexes with the cryo-EM structure of nAChR in complex with nicotine (PDB entry 6cnj; Walsh *et al.*, 2018 ▸) and the sequences of human α4, α7 and β2 subtypes were carried out (Fig. 4 ▸). We considered the orthosteric sites of human heteromeric α4(+)/β2(−) nAChR and homomeric α7 nAChR and first asked how similar the mode of binding of compounds **1**–**3**, and by inference compounds **4**–**6**, might be. The conservation of sequence and structure, in particular considering the aromatic cage of the binding site, suggests that the orientation of the epibatidine analogues is representative of how this compound series would bind to any nAChR. This conclusion is supported by site-directed mutagenesis studies with α7 nAChR, where changes to six residues abolished epibatidine binding (Thompson *et al.*, 2017 ▸). These six residues, which are highly conserved as a group, correspond to Tyr72, Tyr110, Tyr205, Tyr212, Ile123 and Trp164 in *Ac*AChBP (Figs. 2 ▸ and 4 ▸).

The similarity of the amino acids implicated in interactions with the compound set **1**–**6** extends beyond these six residues. On the principal side, Val165 of *Ac*AChBP, which participates in van der Waals interactions with the ligands, aligns with Thr183 and Ser172 in the α4 and α7 subtypes, respectively. In terms of size, these represent conservative substitutions. On loop C, Ser206 is changed to a glutamate in nAChR but the side chain is directed away from the binding site. On the complementary side, Met133 and Ile135 of *Ac*AChBP are Phe144 and Leu146 in nAChR β2 and Gln139 and Leu141 in α7, respectively. At the periphery of the binding site the combination of Val125 and Thr127 in *Ac*AChBP is Val136 and Ser138 in β2 and Asn133 and Leu131 in α7. The noteworthy difference is the Met133Gln substitution, which in α7 nAChR will place a polar side chain near the ligands. It is possible that the latter would provide a steric restriction on the placement of ring B in **1** and **3**, and also that the glutamine could form a hydrogen bond to the carboxamide substituent when binding **2**.

Different ideas have been proposed to explain the activity of agonists and antagonists of pLGICs based on the crystal structures of AChBP–ligand complexes (Taly *et al.*, 2009 ▸; Lemoine *et al.*, 2012 ▸; Bertrand *et al.*, 2015 ▸). The use of *Ac*AChBP provides binding and structural data that inform on affinity and aspects of molecular recognition, but it is important to recognize the limitations of this surrogate (for example it has no transmembrane domains) and exercise caution when considering aspects of channel opening and closing. At least three factors appear to contribute to the distinctive responses of ligands that bind to the orthosteric site of pLGICs. Firstly, residues on the complementary side are the primary determinants of ligand affinity; in particular, by combining the presence of cation–π and van der Waals interactions with the aromatic cage to stabilize the complex. This area of the orthosteric site is well targeted by compounds **1**–**6**; hence, they are high-affinity ligands and their potent antagonist effects on nicotine-induced antinociception at nAChRs (Ondachi *et al.*, 2016 ▸) is likely to be a consequence of being bioavailable and able to outcompete nicotine (Table 2 ▸).

Secondly, the conformation of loop C appears to be relevant. In general, this loop displays three states. There is a fully contracted, closed and clamped conformation when binding small agonists (for example nicotine or epibatidine), a fully extended and open form in the presence of larger antagonist ligands (for example strychnine) and an intermediate state that is stabilized in the presence of partial agonists (for example varenicline). However, the conformation of loop C does not always correspond in such a simple fashion to the pharmacological profile of a ligand. The noteworthy exception is the high-potency antagonist dihydro-β-erythroidine, which induces a loop C conformation similar to that of agonists (Shahsavar *et al.*, 2012 ▸).

In a study of the drug varenicline, Billen *et al.* (2012 ▸) drew attention to a third factor in ligand response. Partial agonists can desensitize pLGICs with the ability to induce opening with higher affinity but with lower efficacy than a full agonist. By targeted site-directed mutagenesis and electrophysiology on α4β2 nAChR they showed that residues on loops D and E, the complementary side of the orthosteric site, contribute to desensitization and channel opening. In particular, inter­actions of β2 nAChR residues Trp82 and Leu146 are important for channel opening. In *Ac*AChBP these correspond to Tyr72 on loop D and Ile135 on loop E (Fig. 4 ▸). These residues are conserved and interact with compounds **1**–**3**, and by implication also with compounds **4**–**6**. Compounds **1** and **3** represent an anomaly, being agonists of α7 nAChR (Ondachi *et al.*, 2016 ▸). The loop C conformations we observe are similar and in an intermediate state. The *Ac*AChBP complex structures with compounds **1** and **3** suggest van der Waals interactions with Val125 and Met133, which are on loop E. These two residues differ in the α7 and β2 nAChR forms (Fig. 4 ▸). In β2 Val125 and Met133 correspond to a valine and a phenyl­alanine, respectively. In the α7 form they are a leucine and glutamine, respectively, and we speculate that different interactions between the ligands and this part of the orthosteric site may be linked to the agonist property of compounds **1** and **3**.

**Figure 5:**
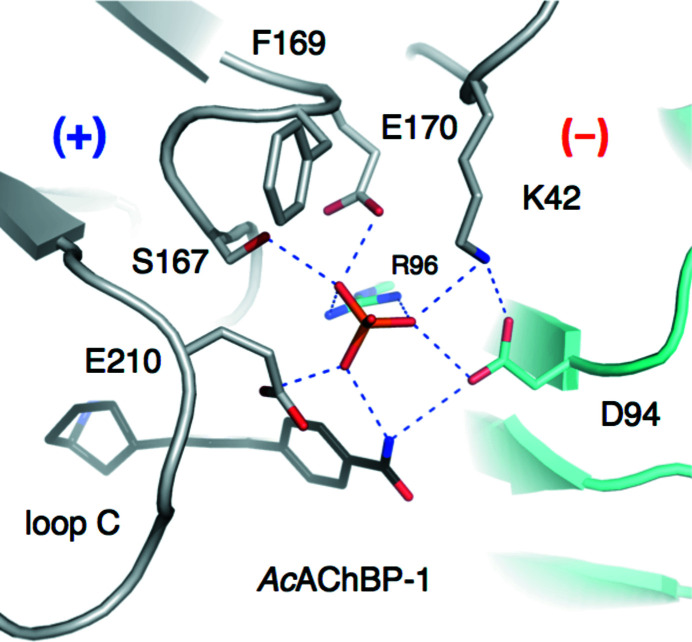
Oxyanion binding at the entrance to the orthosteric site. The polypeptide is shown in ribbon format coloured grey for the principal subunit, which is also marked (+). The complementary side (−) is coloured cyan. Specific residue side chains are shown as sticks with C-atom positions coloured according to the subunit to which they belong, O atoms in red, N atoms in blue, the P atom in orange and C atoms of compound **1** coloured black. Blue dashed lines represent potential hydrogen-bonding inter­actions.

Allosteric control is an important facet of pLGIC function and has been studied extensively in nAChRs (Taly *et al.*, 2014 ▸; Chatzidaki & Millar, 2015 ▸). Of note is the role of Ca^2+^, which increases the affinity for agonists and potentiates their activity on nAChRs, producing an increase in current amplitudes. A number of residues at distinct sites on nAChR structures are implicated in Ca^2+^ binding, and this is indicative of multiple sites of regulation (Galzi *et al.*, 1996 ▸). In this context, the identification of oxyanion binding in two of the structures reported here, involving charged and conserved residues at the subunit interface near the orthosteric site and which bring the key loop C into play, is intriguing (Fig. 5 ▸). These residues have not been previously discussed or investigated in the context of Ca^2+^ binding or allosteric regulation to the best of our knowledge. It may seem counterintuitive to invoke cation binding to a site where an oxyanion is found. However, our crystals were grown in the presence of phosphate and at acidic pH. The bound phosphate may be an artefact of these conditions, but nevertheless draws attention to a group of charged residues that are able to interact with each other or with an ion, which is exactly the type of feature that is likely to be involved in allosteric regulation.

Asp94 and Glu170 in *Ac*AChBP, which as mentioned are likely to be protonated, are replaced by residues which are well established as phosphate-interacting amino acids in nAChR sequences. In human β2, α4 and α7 nAChR, Asp94 corresponds to Lys104, Ser110 and Thr99, respectively, whilst Glu170 corresponds to Glu182, Lys188 and Ser177, respectively. These are adjacent to the strictly conserved Arg96 (Arg110 and Arg101 in β2 and α7, respectively) on the complementary side. From the principal side *Ac*AChBP Glu210, on loop C, aligns with Ser219 in the β2 form, Glu228 in α4 and Glu215 in α7 (Fig. 3 ▸). Note, however, that loop C in the β2 form is potentially very different from those of α4 and α7 (Fig. 4 ▸). Adjacent to Glu210 is Ser167, which corresponds to Asp185 (α4), Gly174 (α7) and Asp179 (β2). It would be too speculative to align Lys42 in *Ac*AChBP with a specific residue in α4 or α7 since it is in a region where, as shown by an overlay with the cryo-EM structure of an nAChR (not shown), conservation is lacking. Nevertheless, the residue types in this location would be compatible with an anion-binding site and such an event may influence the activity of compounds **1**–**6**. This site could represent a point of allosteric control to gating.

## 4. Conclusions

Using *Ac*AChBP as a surrogate of nAChRs, we characterized the binding affinity of a series of epibatidine analogues and were able to determine three crystal structures to inform on protein–ligand interactions. These data may inform the design of new nAChR targeting ligands with defined pharmacological properties. Differing interactions with Tyr212 and Asp94 of *Ac*AChBP appeared to mediate ligand affinities, with interactions at Asp94 potentially also explaining their subtype-selective nAChR pharmacological profiles due to the differing equivalent residues. It may be possible to modify the epibatidine framework, or indeed some other scaffold, to build interactions at a putative ion-binding site to investigate further. Data derived from compounds **1** and **3** suggest that distinction between α4β2 and α7 nAChR forms might involve two residues on loop E, the complementary side of the orthosteric binding site, and it could be instructive to test this hypothesis. Future work will seek to address these issues.

## Supplementary Material

PDB reference: *Ac*AChBP, complex with compound **1**, 6qkk

PDB reference: complex with compound **2**, 6qqp

PDB reference: complex with compound **3**, 6qqo

Supplementary Figures. DOI: 10.1107/S2059798322000754/gi5034sup1.pdf

## Figures and Tables

**Table 1:** Crystallographic statistics for the *Ac*AChBP–ligand complexes Values in parentheses are for the highest resolution shell.

	*Ac*AChBP–**1**	*Ac*AChBP–**2**	*Ac*AChBP–**3**
PDB code	6qkk	6qqp	6qqo
Data collection			
*a*, *b*, *c* (Å)	211.0, 131.6, 131.8	209.5, 136.9, 131.5	209.5, 136.9, 131.5
α, β, γ (°)	90, 102.8, 90	90, 102.7, 90	90, 102.6, 90
Space group	*C*2	*C*2	*C*2
Source	DLS microfocus beamline I24	DLS microfocus beamline I24	DLS microfocus beamline I24
Wavelength (Å)	0.96858	0.96858	0.96858
Subunits per asymmetric unit	10	10	10
Resolution range (Å)	48.83–2.20 (2.24–2.20)	46.82–2.40 (2.44–2.40)	46.40–2.50 (2.54–2.50)
Other ligands	NAG, ethane-1,2-diol, phosphate, oxalate	NAG, ethane-1,2-diol, phosphate	NAG, ethane-1,2-diol, phosphate
Total No. of reflections	611399 (30075)	405440 (19537)	254487 (12673)
Unique reflections	177603 (8766)	139199 (6884)	109552 (5537)
Multiplicity	3.4 (3.4)	2.9 (2.8)	2.3 (2.3)
*R*_merge_	0.172 (0.888)	0.089 (0.510)	0.118 (0.559)
*R*_p.i.m._	0.162 (0.831)	0.088 (0.497)	0.114 (0.539)
Wilson *B* factor (Å^2^)	15.5	29.0	13.6
Completeness (%)	99.9 (98.4)	98.7 (98.7)	89.7 (91.8)
〈*I*/σ(*I*)〉	6.6 (2.0)	8.2 (2.5)	6.9 (2.6)
CC_1/2_	0.95 (0.43)	0.95 (0.65)	0.93 (0.46)
Refinement			
*R*_work_/*R*_free_	0.199/0.228	0.188/0.210	0.193/0.211
No. of reflections for *R*_work_/*R*_free_	168732/8867	131926/6903	104105/5421
Protein residues	2065	2054	2053
No. of ligands	10	10	10
No. of water molecules	1287	924	659
R.m.s.d.s			
Bond lengths (Å)	0.048	0.011	0.012
Angles (°)	0.97	1.49	1.56
Ramachandran plot			
Residues in favoured regions	2002	1981	1964
Residues in allowed regions	35	30	35
Residues in outlier regions	0	1	0
Mean *B* factors (Å^2^)			
Protein atoms per subunit	18.4/15.7/17.3/19.2/17.3/18.4/20.5/22.2/22.6/19.2	34.2/30.7/33.1/29.9/32.2/37.7/40.4/41.2/34.4/34.0	19.7/17.3/19.1/17.2/18.9/22.2/24.0/23.6/19.3/19.6
Water molecules	22.2	36.1	17.1
Ligand	14.5/14.2/16.4/14.8/15.3/16.8/17.8/20.7/18.1/18.1	32.8/31.0/28.7/26.1/35.4/38.9/43.7/40.7/30.2/35.6	17.9/19.8/22.0/14.4/26.8/28.1/35.1/27.0/16.3/29.0
NAG	68.7	112.7	87.6
Ethane-1,2-diol	34.4	46.7	36.6
Phosphate	41.0	—	54.9
Oxalate	41.5	—	—

**Table 2:** Ligand-binding properties Values are given with standard errors.

Ligand	*Ac*AChBP, *K*_d_ (n*M*)	α_4_β_2_ nAChR, *K*_i_ † (n*M*)
**1**	9.8 ± 0.03	0.12 ± 0.020
**2**	60.0 ± 0.14	0.28 ± 0.010
**3**	29.0 ± 0.05	0.94 ± 0.070
**4**	33.0 ± 0.08	0.07 ± 0.002
**5**	30.0 ± 0.06	0.28 ± 0.030
**6**	9.8 ± 0.01	0.67 ± 0.280
Epibatidine	14.0‡	0.02 ± 0.001
Varenicline	342.0§	0.12 ± 0.002
Nicotine	835.0§	0.95¶

† [^3^H]-Epibatidine competition assay, *K*_d_ = 0.02 n*M* (Ondachi *et al.*, 2016 ▸).

‡ Tryptophan fluorescence-quenching assay (Hansen *et al.*, 2005 ▸).

§ Isothermal titration calorimetry (Rucktooa *et al.*, 2012 ▸).

¶ [^3^H]-Nicotine competition assay (Coe *et al.*, 2005 ▸).
