# Supplementary material for: Interactions between 2′-fluoro-(carbamoyl­pyridinyl)deschloroepibatidine analogues and acetylcholine-binding protein inform on potent antagonist activity against nicotinic receptors
Source: Acta Crystallogr D Struct Biol. 2022 Feb 21;78(Pt 3):353–62. doi: 10.1107/S2059798322000754 (PMC8900824; doi:10.1107/S2059798322000754)
Supplement: Supplementary file 1 [file d-78-00353-sup1.pdf]

STRUCTURAL
BIOLOGY

Volume 78 (2022)

Supporting information for article:

Interactions between 2′-fluoro-(carbamoylpyridinyl)deschloroepibatidine analogues and acetylcholine-binding protein inform on potent antagonist activity against nicotinic receptors

Renata V. Bueno, Samuel Davis, Alice Dawson, Pauline W. Ondachi, F. Ivy Carroll and William N. Hunter

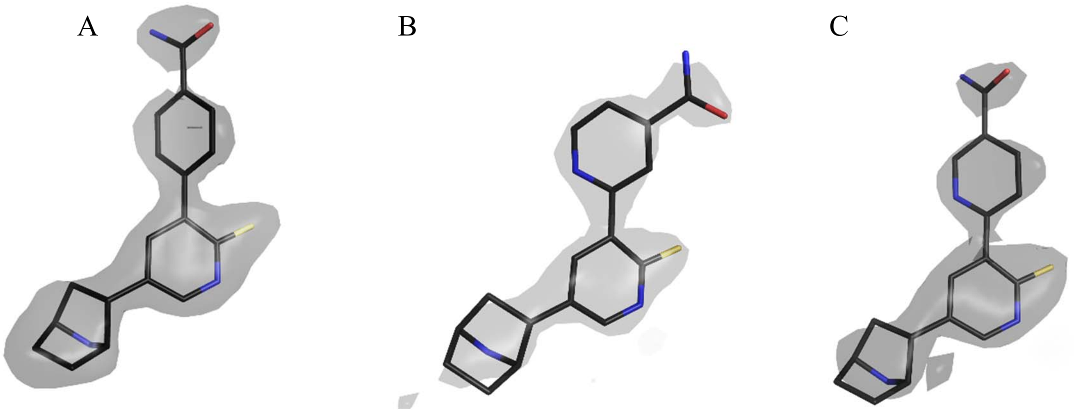

Figure S1 Representative F_o-F_c omit maps of A. compound 1, B. 2 and C. 3 contoured at 4.0 r.m.s.d. The bulk solvent correction was disabled and the ligand atoms were removed from the model prior to the calculation of F_c. Ligands are depicted as sticks colored according to atom type: C black, N blue, O red, F pale yellow. 98 should be 9.8 below

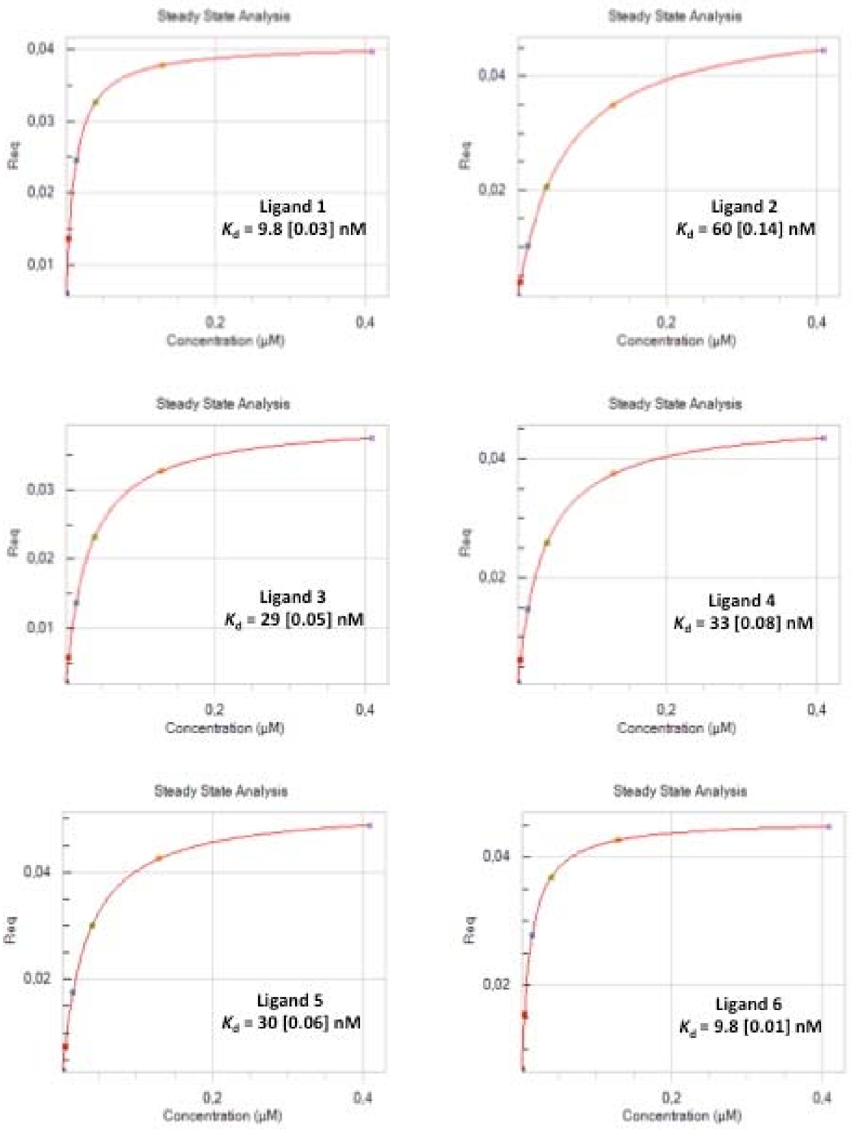

Figure S2 BLI plots.
